# The UK National Neonatal Research Database: using neonatal data for research, quality improvement and more

**DOI:** 10.1136/archdischild-2015-309928

**Published:** 2016-03-11

**Authors:** C Gale, I Morris

**Affiliations:** 1 Imperial Clinical Trials Unit, Imperial College London, London, UK; 2 Neonatal unit, University Hospital Wales, Cardiff, UK

**Keywords:** Epidemiology, Health services research, Information Technology, Neonatology

## Introduction

Electronic data are increasingly recorded in clinical practice. Just as advances in genetics have gradually led to clinical benefit^1^ so too are ‘big data’ bringing tangible advances to patient care.^2^

The UK has a long history of using electronic neonatal data for research and is now in the enviable position of having electronic patient data on all admissions to National Health Service (NHS) neonatal units in England, Wales and Scotland. This national resource, the National Neonatal Research Database (NNRD), is available for research, audit, benchmarking and quality improvement. Here, we provide an overview of how data entered into an electronic system (Badger.net; Clevermed Ltd) as a component of day-to-day care, are used to form the NNRD and how this can be used by health professionals.

## A brief history of neonatal databases

For over 25 years, neonatal data have been collected in regional databases such as The Neonatal Survey. The availability of national neonatal data is, however, a relatively recent phenomenon in the UK and internationally. In the UK the use of electronic patient records shared across neonatal units began in 2004 as a regional initiative. This platform, Badger.net, subsequently expanded nationwide; it is now used by many neonatal units to plan services and record activity for payment by NHS England.

In 2007, the Neonatal Data Analysis Unit (NDAU) was established at the Chelsea and Westminster Hospital campus of Imperial College London to improve the quality of electronic clinical data and promote their use to support neonatal services and facilitate research. Data entered onto the Badger.net system are extracted at intervals, undergo quality assurance procedures, are anonymised and entered into the NNRD (box 1).

Box 1 **Data management services performed by the Neonatal Data Analysis Unit (NDAU) on Badger.net data prior to forming the National Neonatal Research Database (NNRD)**

- Separate patient identifiers into a discrete relational database
- Merge data packets to create a single file for each patient for each neonatal unit episode
- Link patient episodes across neonatal units (transfers) to create single linked episode file for each patient to discharge or death
- Identify and flag missing, inconsistent and out-of-range data for feedback to Neonatal Units
- Link NNRD to Hospital Episode Statistics and Office for National Statistics data

## How the NDAU and the NNRD work

**Figure 1:**
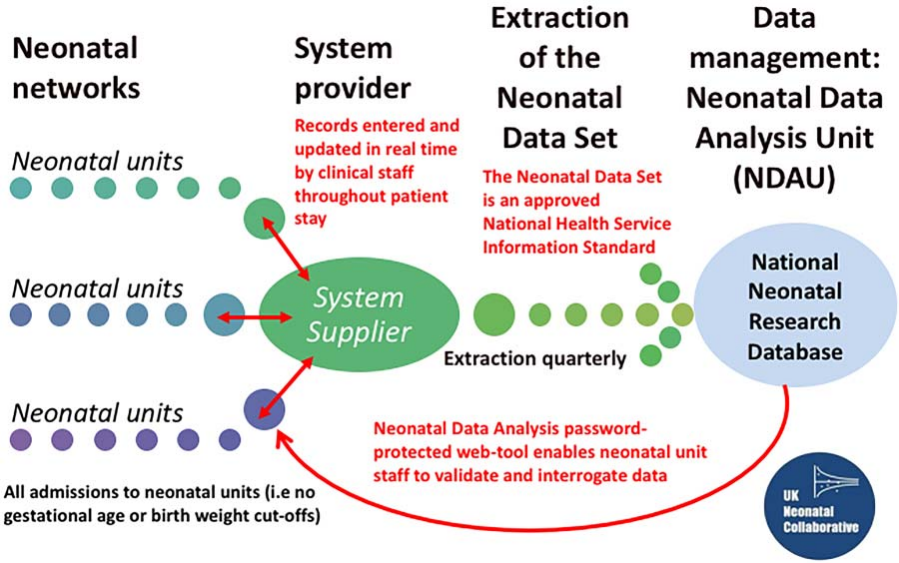
Data flows to the National Neonatal Research Database. (Courtesy of Mr Eugene Statnikov and Professor Neena Modi).

All neonatal units that contribute data to the NNRD form the UK Neonatal Collaborative (currently 100% of neonatal units in England, Wales and Scotland). NNRD data originate from information entered by clinicians (usually trainees) and nursing staff onto the Bager.net platform at the point of care. Approximately 400 predefined data items, the Neonatal Data Set, are extracted quarterly from these electronic patient records to form the NNRD (figure 1). The Neonatal Data Set is an approved NHS Information Standard hence any neonatal electronic system must be able to capture these items. To date the NNRD holds data on approximately 500 000 patients with 20 000 added quarterly. Data include ICD10 codes and mapping to Systematized Nomenclature of Medicine–Clinical Terms (SNOMED-CT)^3^ is underway.

The NNRD is a national Research Ethics Committee approved database; Caldicott Guardians and Neonatal Lead Clinicians of all contributing Trusts have approved the use of the NNRD for health services evaluations and authorised research. Parents are informed about the NNRD and can opt out at any time. Neonatal electronic patient record data can be used by health professionals in a variety of ways.

## Using neonatal data locally

The electronic platform (Badger.net) provides ready access to local data by local clinicians. These may be used for audits, service evaluations and quality improvement projects; examples include:

1. Identification of procedures (eg, percutaneous central lines).
2. Identification of complications (eg, blood stream infections).
3. Describing resource usage (eg, duration of stay).
4. Production of activity reports.

Many neonatal units have staff able to assist in using the electronic platform in this way; additionally Clevermed (the commercial provider of the Badger.net platform) can provide support: http://www.clevermed.com.

## Using the NNRD

The NNRD is a national resource to support regional and national quality improvement, audit, benchmarking and research. Data held in the NNRD cover the whole clinical stay for each infant, linking all episodes of care. The NDAU can provide data extraction, analysis and statistical support; however, because the NDAU receives no core funding to maintain the NNRD, a charge is necessary to cover data transfer, extraction, cleaning, storage and any analyses requested. Examples of NNRD data use include:

1. Evaluating the national reorganisation of neonatal services into managed clinical networks.^4^
2. Describing longitudinal growth among preterm infants.^5^
3. Evaluating the impact of a regional quality improvement project.^6^

Further work seeks to establish the use of the NNRD for randomised clinical trials.^7^

If clinicians wish to use NNRD data for a regional, national or international project or in research, the NDAU can be contacted at https://www1.imperial.ac.uk/departmentofmedicine/divisions/infectiousdiseases/paediatrics/neonatalmedicine/ndau/. Costs of using the NNRD can be built into funding applications so we advise contacting the NDAU early in the development of a proposed study.

## Summary

Data entered by UK neonatal professionals into the Badger.net platform are used to form a unique resource, the NNRD. As a result of the diligence taken by the numerous (often trainee) doctors and nurses who enter data every day, the NNRD forms one of the most detailed, accurate and complete population level neonatal datasets worldwide. The NNRD can be used to support local, regional, national and international work ranging from audit to observational and interventional research. Paediatricians and neonatologists are ideally placed to identify research questions with the potential to improve neonatal care; the NDAU and the NNRD can support health professionals in undertaking such initiatives.
